# Ecosystem carbon emissions from 2015 forest fires in interior Alaska

**DOI:** 10.1186/s13021-017-0090-0

**Published:** 2018-01-08

**Authors:** Christopher Potter

**Affiliations:** NASA Ames Research Center, Moffett Field, Mountain View, CA USA

**Keywords:** Wildfire, Carbon emission, Alaska, Boreal forest, Soil carbon, Landsat

## Abstract

**Background:**

In the summer of 2015, hundreds of wildfires burned across the state of Alaska, and consumed more than 1.6 million ha of boreal forest and wetlands in the Yukon–Koyukuk region. Mapping of 113 large wildfires using Landsat satellite images from before and after 2015 indicated that nearly 60% of this area was burned at moderate-to-high severity levels. Field measurements near the town of Tanana on the Yukon River were carried out in July of 2017 in both unburned and 2015 burned forested areas (nearly adjacent to one-another) to visually verify locations of different Landsat burn severity classes (low, moderate, or high; LBS, MBS, HBS).

**Results:**

Field measurements indicated that the loss of surface organic layers in boreal ecosystem fires is a major factor determining post-fire soil temperature changes, depth of thawing, and carbon losses from the mineral topsoil layer. Measurements in forest sites showed that soil temperature profiles to 30 cm depth at burned forest sites were higher by an average of 8–10 °C compared to unburned forest sites. Sampling and laboratory analysis indicated a 65% reduction in soil carbon content and a 58% reduction in soil nitrogen content in severely burned sample sites compared to soil mineral samples from nearby unburned spruce forests.

**Conclusions:**

Combined with nearly unprecedented forest areas severely burned in the Interior region of Alaska in 2015, total ecosystem fire-related losses of carbon to the atmosphere exceeded most previous estimates for the state, owing mainly to inclusion of potential “mass wasting” and decomposition in the mineral soil carbon layer in the 2 years following these forest fires.

## Background

The 2015 fire season in Alaska resulted in the second highest acreage burned for the state in a single year. In mid-June 2015, nearly 300 fire starts were reported within 1 week, a consequence of over 61,000 detected lightning strikes during the period [2]. As of mid-September, a total of 2.1 million ha (5 million acres) had burned statewide in over 700 separate wildfires. A relatively low snowpack across southern Alaska, compounded by a warm, dry spring, resulted in extremely burnable fuels [2]. Following one of the wettest summers on record in 2014, Alaska’s intense fire season of 2015 was extreme by most historical standards.

Over the past 50 years, there has been an increase in the frequency and severity of boreal forest wildfires in Alaska [17]. During the 2000s, an average of 767,000 ha per year were burned statewide, 50% higher than in any previous decade since the 1940s. Deeper burning of surface organic layers in black spruce (*Picea mariana* (Mill.) BSP) forests has occurred during late growing-season fires and on more well-drained sites [19].

Simulation modeling studies of carbon storage for the state of Alaska have estimated that terrestrial ecosystems have been a net carbon sink (from the atmosphere) of between 5 and 12 Tg C (1 Tg = 10^12^ g) year^−1^ in the 1980s, and between 0 and 10 Tg C year^−1^ during the 1990s and 2000s [6, 34, 35]. Such a wide range of estimates for ecosystem carbon balance in Alaska has resulted, in part, from large uncertainties in region-wide wildfire emissions of carbon, which have been reported over a range of 14–81 Tg C year^−1^ (Table 1). The majority of previous carbon emission studies for Alaska to date have relied on measurements of aboveground (tree) biomass and changes in surface organic layer carbon pools, while generally not including changes in mineral topsoil carbon pools after large-scale burning of the surface layers at moderate and high severity levels.

**Table 1:** Previous estimates of regional carbon emissions from forest fires in Interior Alaska

Region	Year(s)	Tg C year^−1^	Error (±)	References
Yukon River Basin, Alaska	2004	81	13.6	Tan et al. [26]
Alaska boreal forests	2000–2009	14	0.6	Turetsky et al. [28]
Alaska boreal forests	2004	69		Veraverbeke et al. [31]
Alaska boreal forests and wetlands	1950–2009	39		McGuire et al. [23]

The objectives of this study were to (1) conduct field validation and statistical comparisons of the burned index rankings of 2015 wildfire areas near Tanana, Alaska to Landsat burn severity classes mapped (post-fires) in 2015 and 2016, and (2) estimate total ecosystem (live biomass and mineral topsoil) carbon emissions from the 2015 wildfires across the Yukon–Koyukuk forest region. This work was undertaken as a contribution to the NASA Arctic Boreal Vulnerability Experiment (ABoVE) field campaign, chiefly to better understand changes in related hydrologic and biogeochemical mechanisms in the years following boreal forest wildfires. One of the major questions being addressed by ABoVE is “What processes are controlling changes in boreal-arctic land cover properties and what are the impacts of these changes?”.

## Methods

### Study area

**Fig. 1:**
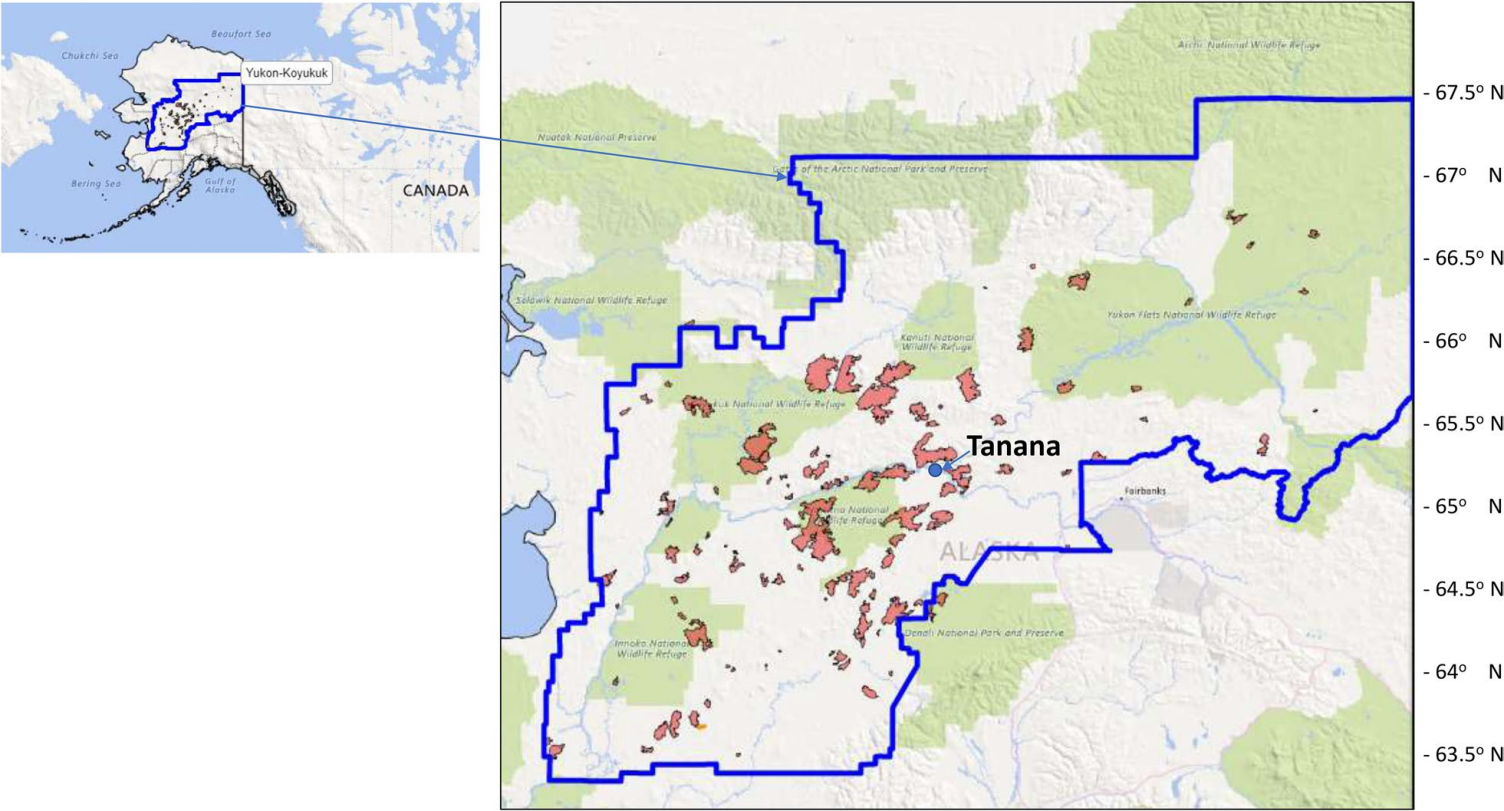
Wildfires from 2015 analyzed for Landsat RdNBR classes in the Yukon–Kukukuk region of Alaska from the MTBS

The area studied was boreal forest of the Yukon–Koyukuk region of Alaska (Fig. 1). Field measurements were carried out in forests of various states of disturbance from 2015 wildfires surrounding the confluence of the Yukon and Tanana Rivers (near 65°8′N latitude, 152°27′W longitude), about 200 km west of Fairbanks, Alaska. Mean annual temperature over much of Interior Alaska is well below freezing, which accounts for a permafrost distribution that is commonly continuous, except in the southern portion of the region [8]. The climate near Tanana is characterized by mean monthly temperature variations between – 27 and 22 °C, and a mean annual precipitation total of 29 cm, 11 cm of which falls as snow (data available online at http://www.usclimatedata.com).

Forests in the study area are predominately black spruce on wetter soils and white spruce (*Picea glauca*) on drier soils, described by [33] as follows: Open black spruce forest description—Total arboreal cover is between 25 and 60%. Paper birch (*Betula papyrifera*) may be present in small amounts. The trees tend to be small; the largest trees are about 5–10 cm in diameter and 6–10 m tall. A well-developed tall shrub layer dominated by dwarf birch (*Betula glandulosa*) 1–2 m high often is present. Other tall shrubs locally important on moist sites include *Alnus crispa*, *A. sinuata, Salix *spp., and *Rosa acicularis*. A low shrub layer usually is present and consists primarily of some combination of *Vaccinium uliginosum, V. vitis*-*idaea, Potentilla fruticosa, Arctostaphylos rubra, Empetrum nigrum,* and *Ledum* spp. The moss layer is continuous or nearly so and dominated by a combination of *Hylocomium splendens, Pleurozium schreberi, Polytrichum* spp., and *Dicranum* spp. Lichens such as *Cladonia* spp. are important on some sites.

Closed white spruce forest description—the closed white spruce forest type represents the best developed, most productive forests in Alaska. The over-story canopy cover, usually entirely white spruce but occasionally with either scattered paper birch or balsam poplar (*Populus balsamifera*) can range from 60 to 100%. On the best sites, trees reach 30 m in height. A well-developed moss layer consisting primarily of the feathermosses *Hylocomium splendens, Pleurozium schreberi*, and less commonly, *Rhytidialdelphus triquetrus* is characteristic of these stands. Herbaceous growth is usually sparse but horsetails, primarily *Equisetum sylvaticum* and *E. arvense*, may provide as much as 50% cover in flood-plain stands. Other forbs include *Pyrola* spp., *Linnaea borealis, Geocaulon lividum, Mertensia paniculata*, and *Goodyera repens.*

The Soil Survey for the Upper Tanana Area (USDA, 1999) described the soil types most representative of our study sites, namely Goldstream peat on 0–3% slopes, alluvial plains, and moraines. These soils are further characterized in this survey as having an organic surface mat 20–40 cm thick, on top of a dark gray silt loam 15–30 cm deep. These soils are very poorly drained, with permafrost as the root-restricting feature at 25–50 cm depth.

### Landsat burn severity classes

Digital maps of burn severity classes at 30-m spatial resolution for wildfires in 2015 across the Yukon–Koyukuk region of Alaska were obtained from the Monitoring Trends in Burn Severity (MTBS) project, which has consistently mapped fires greater than 1000 acres across the United States from 1984 to the present [9]. MTBS is conducted through a partnership between the U.S. Geological Survey (USGS) National Center for Earth Resources Observation and Science (EROS) and the USDA Forest Service.

The normalized burn ratio (NBR) index was first calculated using approximately one-year pre-fire and post-fire images from the near infrared (NIR) and shortwave infrared (SWIR) bands of the Landsat sensors.

$${\rm NBR} = ({\rm NIR} - {\rm SWIR})/({\rm NIR} + {\rm SWIR})$$

Pre- and post-fire NBR images were next differenced for each Landsat scene pair to generate the Relative dNBR.

$${\rm RdNBR} = [({\rm NBRpre}{\text{-}}{\rm fire} - {\rm NBRpost}{\text{-}}{\rm fire})]/\sqrt {{\rm ABS}\,({\rm NBRpre}{\text{-}}{\rm fire})}$$

RdNBR severity classes of low, moderate, and high potentially cover a range of − 500 to + 1200 over burned land surfaces. Positive RdNBR values represent a decrease in vegetation cover and a higher burn severity, while negative values would represent an increase in live vegetation cover following the fire event.

### Burn index estimation

**Fig. 2:**
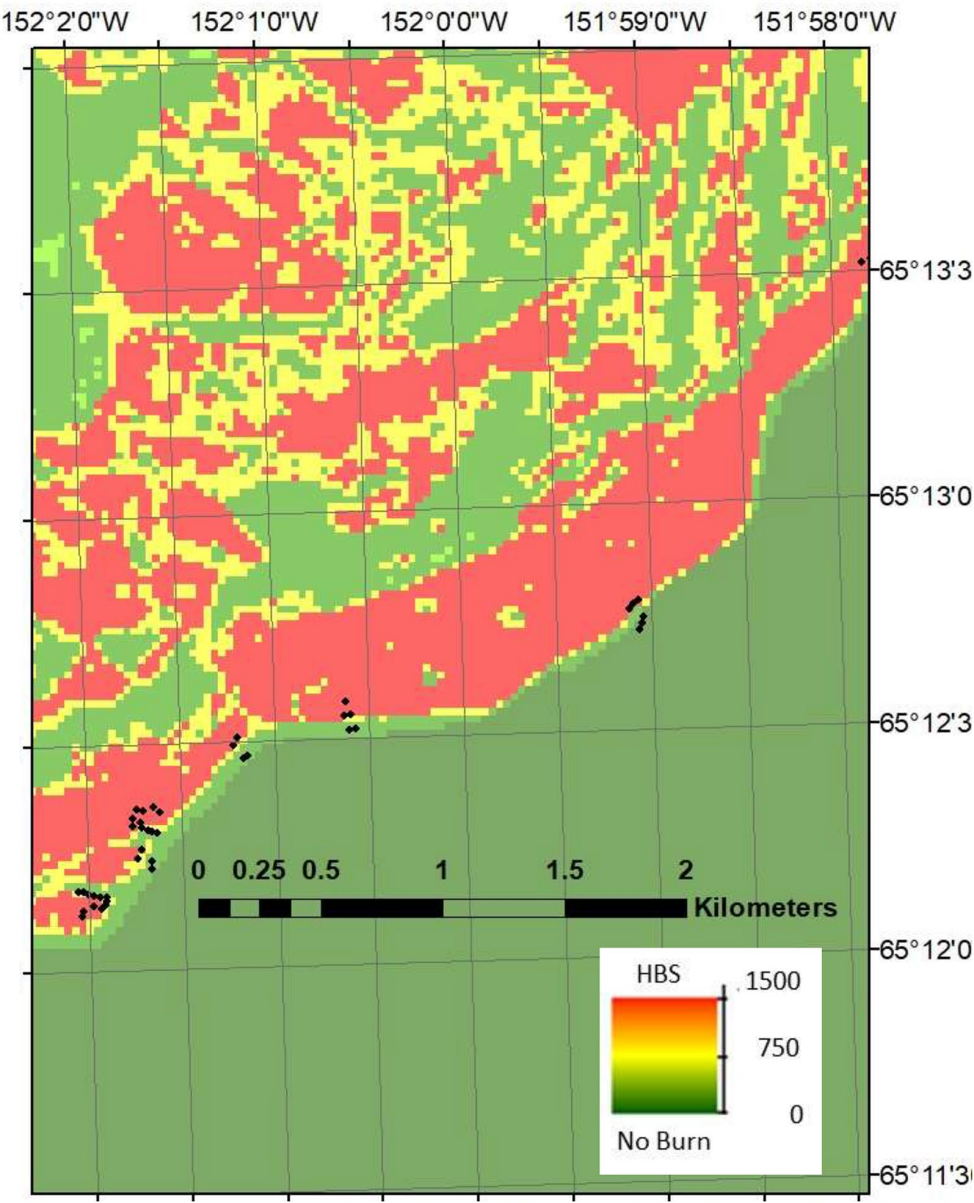
Measurement sites for CBI estimation and soil attributes within the Spicer Creek Fire’s Landsat RdNBR classes

In July 2017, burned areas and adjacent unburned forest stands were surveyed along and within the boundaries of the Tozi-Spicer Creek Fire and the Blind River-Bering Creek Fires on either bank of the Yukon River near Tanana (Fig. 2). Following the Composite Burn Index (CBI) protocol from Key and Benson [20], as customized for forests of Alaska [3], we made ocular estimates at each soil sampling site of the degree of change caused by 2015 wildfire within five forest strata: (1) substrate layer, (2) low vegetation less than 1-m tall, (3) tall shrubs/sapling trees 1–2 m tall, (4) intermediate trees 2–8 m tall, and (5) large trees > 8 m tall. Within each stratum, four to five variables were scored to generate a CBI ranking between 0 and 3 for the level of burn severity. All live and dead plant species were noted and photographed at each forest site visited.

### Soil measurements and sampling

At each sampling site near Tanana, the surface organic layer was excavated in July 2017 to create 30 cm depth soil pits. True color (RGB) and thermal infra-red (TIR) images of all excavated soil pits were collected using a FLIR Series C2 hand-held camera (with an object range of – 10 to 150 °C), recording 320 × 240 pixels per image. All TIR image data was collected over short time window (mid-day hours of 10 a.m. to 2 p.m.) on 5 consecutive days in July 2017 during which air temperature was highly constant and no rainfall events occurred.

At least 500 g of mineral soil sample was collected, starting at 10 cm depth (below the bottom level of the surface organic layer) down to 30 cm mineral soil depth from each pit, sealed in ziplock plastic bags, and shipped to the Oregon State University Crop and Soil Science Central Analytical Laboratory for analysis of carbon and nitrogen content by the total elemental combustion technique. A total of 19 unburned and 19 burned forest soils were excavated to a depth of 30 cm in the soil pits and sampled in this manner.

To verify soil pit TIR imaging patterns with depth, soil temperature was measured using a ThermCo digital thermometer with a 7-cm stainless steel probe inserted into the organic layer ground cover, and at 10 and 30 cm soil depths.

### Statistical analysis

Linear least squares regression was used to test for significant correlation relationships between burn severity attributes. Tests of statistical significance between unburned and burned site attributes were carried out using the two-sample Kolmogorov–Smirnov (K–S) test, a nonparametric method that compares the cumulative distributions of two data sets [21]. The K–S difference test does not assume that data were sampled from Gaussian distributions (nor any other defined distributions), nor can its results be affected by changing data ranks or by numerical (e.g., logarithm) transformations. The K–S test reports the maximum difference between the two cumulative distributions, and calculates a probability (*p*) value from that difference and the group sample sizes. It tests the null hypothesis that both groups were sampled from populations with identical distributions according to different medians, variances, or outliers. If the K–S *p* value is small (i.e., < 0.05), it can be concluded that the two groups were sampled from populations with significantly different distributions.

## Results

### CBI versus RdNBR

**Fig. 3:**
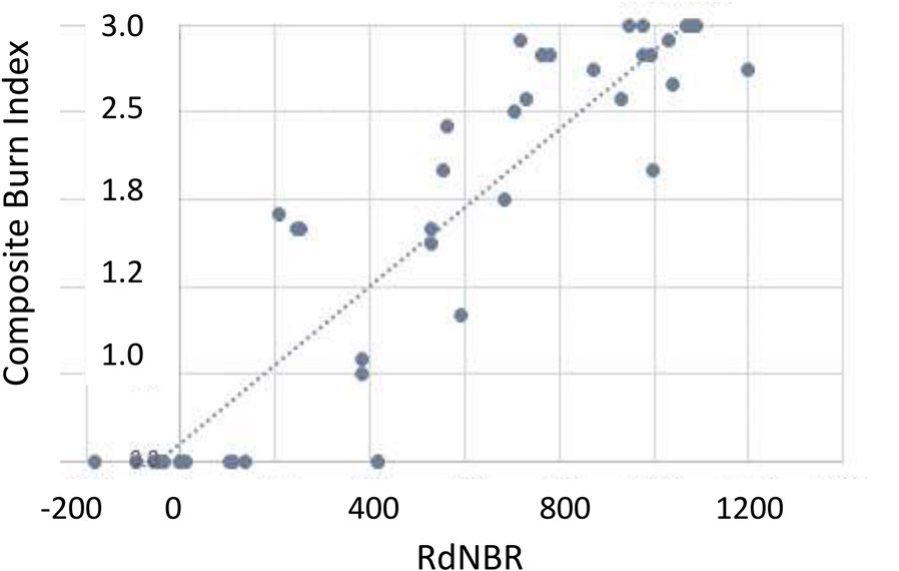
Correlation of the Landsat RdNBR (from 2016) with CBI estimates for forest sites surveyed near Tanana in July 2017

Field surveys across a total of 48 unburned and burned (in 2015) forest sites near Tanana showed that the measured CBI was significantly correlated (at *p* < 0.01, *R*^2^ = 0.85) with the Landsat RdNBR from both 2015 and 2016 post-fire images (Fig. 3). An observed CBI value of 3.0, indicating complete consumption of all pre-fire forest (strata) biomass during the 2015 fires, corresponded to a Landsat RdNBR value of about 1000 and the most extreme HBS post-fire conditions.

### Plant growth in HBS areas

At all sites recorded with a CBI value greater than 2.0, there was no observed regrowth in July 2017 of any shrub or tree species that was observed growing in any the unburned spruce forest sites (CBI = 0), as listed in the study area description above. At all HBS locations we surveyed, the substrate layer was comprised of entirely dead (charred blackened) moss and lichen cover. Occasional hummocks 50 cm deep (or deeper) and several meters in length of dead moss layer were encountered in transect crossings of these HBS areas. The low vegetation stratum (< 1-m tall) at all HBS areas visited was comprised of relatively sparse coverage of fireweed (*Chamaenerion angustifolium*), horsetails, and mixed grasses. Ground cover plant species commonly seen in unburned forest locations, but not seen regrowing in HBS locations in 2017, were bog blueberry (*Vaccinium uliginosum*) and highbush cranberry *(Vibernum edule).*

### Differences in surface organic layer thickness and temperature

**Fig. 4:**
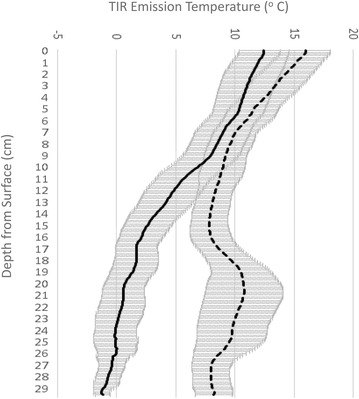
Average TIR temperature profiles for 19 burned (CBI > 2; dashed line) and 19 unburned (CBI = 0; solid line) soil pits excavated to 30 cm depth. Error bars show 2 standard errors of the mean

Visual evaluation of paired (unburned and burned) true color photos of organic soil layer thickness revealed that severely burned forest sites (CBI > 2) had lost between 5 and 10 cm of the thick live moss and lichen cover observed at every unburned forest site surveyed in 2017. By comparisons of soil TIR temperature profiles, we measured a significant separation (*p* < 0.05) in averaged soil temperature profiles between unburned and severely burned forest sites (CBI > 2), beginning around 14 cm soil depth (Fig. 4). The profile temperatures commonly stabilized at between 8 and 12 °C in HBS site soils below about 15 cm depth from the top of the remaining organic surface layer. In contrast, at unburned forest sites, measured TIR temperatures continued to decline gradually to below 0 °C at a typical soil depth of 25 cm from the top of the thick (10-cm) intact organic surface layer of moss and lichen cover. Averages of pit profile data showed the higher temperatures of 5–8 °C at 30 cm depth in the HBS soil profiles, compared to consistently freezing temperatures measured at bottom of the 30-cm deep unburned site profiles.

These TIR imaging differences were confirmed by soil probe measurements, which showed that mean soil temperatures recorded at 10 cm depth were significantly greater (*p* < 0.001) in burned forest sites (CBI > 2, n = 19) at 8.1 °C compared to unburned sites (CBI = 0, n = 19) with a mean value of 3.0 °C. Furthermore, mean soil temperatures recorded at 30 cm depth were significantly greater (*p* < 0.001) in burned forest sites (CBI > 2) at 6.5 °C compared to unburned sites (CBI = 0) with a mean value of 0 °C.

### Differences in mineral soil carbon and nitrogen

There was a significant difference (*p* < 0.05) in both surface mineral soil carbon and nitrogen content from unburned (CBI = 0) and severely burned (CBI > 2) forest sites near Tanana Alaska in July 2017 (Table 2). On average, there was a 65% reduction in soil carbon content and a 58% reduction in soil nitrogen content in severely burned sample sites compared to soil mineral samples from nearby unburned spruce forests. This resulted in the soil mineral C:N ratio decreasing by 20% in severely burned sample sites, due to the higher relative loss of soil carbon over soil nitrogen during or after the 2015 wildfires.

**Table 2:** Surface mineral soil carbon and nitrogen content from unburned (CBI = 0) and severely burned (CBI > 2) forest sites in 2015 near Tanana Alaska

	C (% sample dry weight)	N (% sample dry weight)	C/N ratio
Mean CBI = 0	12.56	0.64	19.21
Mean CBI > 2	4.38	0.27	15.39
2SE CBI = 0	4.76	0.25	1.85
2SE CBI > 2	1.88	0.11	1.15
Min CBI = 0	1.14	0.15	7.60
Min CBI > 2	0.90	0.09	10.00
Max CBI = 0	37.74	1.93	25.06
Max CBI > 2	18.45	1.08	18.38
K–S test *p*	< 0.01	< 0.05	< 0.01

2SE indicate two standard errors of the mean

These measured fractional changes in soil C and N content of unburned and severely burned forests, adjusted by previous soil bulk density measurements from forest sites across Alaska (Table 3), resulted in average estimated loss of carbon since the 2015 wildfires equal to 15.6 kg C m^−2.^from the mineral soil layer (sampled to 30-cm depth). Carbon loss from the severe burning of live moss and the surface organic layers together were estimated at slightly more than 9 kg C m^−2^.

**Table 3:** Live moss, surface organic layer, and soil carbon content (to 30 cm depth) estimated for unburned and severely burned (since 2015) forests near Tanana, based on previous bulk density measurements from forest sites across Alaska and percent mineral soil carbon changes from Table 1. Bulk density (g cm^−3^)

Horizon	Site 1	Site 2a	Site 2b	Site 3	Mean	Kg C m^−2^ unburned	Kg C m^−2^ burned	Kg C m^−2^ difference
Moss				0.02	0.02	1.0	0.5	0.5^a^
A	0.40	0.30	0.50		0.35	17.6	8.8	8.8^a^
B	0.75	0.52			0.64	23.9	8.3	15.6

Bulk density measurements of surface organic layer (A) and mineral horizon (B)

Site 1 [5]

Site 2 [14]

Site 3 [25]

^a^ Carbon difference between unburned and severely burned sites for live moss and A horizon was assumed to be 50% by weight [26]

### Landsat burn severity areas for 2015

Compilation of burn severity class areas for 113 wildfires mapped in 2015 from the MTBS project (Fig. 1) showed a total of 1.64 million ha burned across the Yukon–Koyukuk region of Alaska (Table 4), with averages of 30 and 27% at MBS and HBS fraction per fire, respectively. Total regional 2015 burned areas were estimated at 0.47 million ha MBS and 0.52 million ha HBS. Among the largest of the 2015 fires, in excess of 70,000 ha total area burned, were the Middle Yukon and Tanana Area Fires, the latter of which was mapped at 48% HBS area. The majority of these largest Alaska forest wildfires in 2015 were located between 64.5° and 66°N latitude.

**Table 4:** List of wildfires (greater than 10,000 ha) in the Yukon–Koyukuk region of Alaska in 2015

Fire name	HUC4 name	Latitude	Hectares	%MBS	%HBS
Middle Yukon Fires	Nowitna River	64.550	164,890	26	31
Tobatokh	Melozitna River	65.760	89,117	18	49
Isahultila	Koyukuk Flats	66.050	78,020	31	24
Holtnakatna	Dulbi River	65.393	72,949	34	16
Tanana Area Fires	Ramparts to Ruby	65.318	71,590	26	48
Rock	Koyukuk Flats	66.044	57,895	31	45
Big Mud River 1	Nowitna River	64.670	57,069	30	44
Munsatli 2	North Fork Kuskokwim River	63.726	50,455	27	51
Blazo	Lower Innoko River	63.479	50,175	38	9
Sushgitit Hills	Kanuti River	66.051	47,887	24	48
Torment Creek	Kanuti River	65.943	43,304	26	27
Sea	Nowitna River	64.013	40,745	27	35
3 day	Huslia River	65.741	36,420	38	31
Hay Slough	Lower Tanana River	65.034	34,887	29	20
Dulbi River	Dulbi River	65.176	34,389	24	4
Blind River	Ramparts to Ruby	65.109	34,087	32	32
Bering Creek	Ramparts to Ruby	65.014	30,874	34	34
Rungun Creek	North Fork Kuskokwim River	63.565	25,315	26	46
West Fork	Yukon Flats	66.366	24,990	37	18
Iditarod River	Lower Innoko River	62.549	24,644	21	5
Lloyd	Lower Tanana River	64.668	22,890	24	56
Hardpac Creek	Yukon Flats	66.904	20,974	21	53
Carlson Lake	Kantishna River	63.764	19,502	45	8
Lower Reindeer Peak	Lower Innoko River	62.481	19,265	24	21
Old Woman	Unalakleet	64.043	18,812	32	35
Holonada	Tozitna River	65.693	18,346	25	9
Stuyahok River	Anvik to Pilot Station	62.251	18,308	32	21
Sethkokna	Nowitna River	64.258	15,867	28	45
Yukon Creek	Galena	64.300	15,794	29	6
Glacier	Melozitna River	65.129	15,334	35	41
Harper Bend	Lower Tanana River	64.938	15,215	29	44
Nulato	Galena	64.818	14,458	33	26
Hamlin Creek	Ramparts	65.924	14,095	26	49
Hickey Creek	Upper Innoko River	62.600	13,635	23	54
Deepbank Creek	Farewell Lake	62.886	13,386	39	48
Birch Creek 2	Birch-Beaver Creeks	65.372	13,257	28	8
Our Creek	Nowitna River	63.964	12,796	29	48
Aggie Creek	Tolovana River	65.247	12,498	21	24
Lawson	Nowitna River	64.433	12,005	25	45
Soda Creek	North Fork Kuskokwim River	63.246	10,424	21	53
	Sum for all 113 fires		1,635,293		
	Mean		14,472	30	27
	Standard deviation		23,101	9	18
	Maximum		164,890	57	65
	Minimum		453	10	1

### Regional carbon losses from 2015 wildfires

Based on the total MBS and HBS forest areas consumed in 2015 across the Yukon–Koyukuk region (from Table 3), plus the organic layer carbon fractions consumed in MBS and HBS areas from [26], and the estimated loss of carbon since the 2015 wildfires from the mineral soil layer and the live moss and surface organic layers (from Table 2), it was determined that 154 Tg C were lost following the wildfires in interior Alaska in 2015. Mineral soil losses (and surface organic layer carbon emission totals from combustion) did not include the emission from combustion of aboveground forest biomass, which, based on average area-based carbon losses reported by Tan et al. [26] of 2.23 kg C m^−2^, would have added 8.7 Tg C in Alaska wildfire emissions in 2015.

## Discussion

The exceptionally warm and dry conditions leading up to the summer of 2015 were followed by the largest wildfires recorded in decades in interior Alaska. Our estimate of the depth to which MBS and HBS wildfires had burned into and completely consumed surface organic moss layers during the 2015 Tanana fires was between 5 and 10 cm. This burn depth estimate was confirmed using the relationship reported by Harden et al. [11], that for every centimeter of organic mat thickness in boreal forests, soil temperature under the organic layer remained about 0.5 °C cooler during summer months. The difference (increase) we measured in average temperature at 10 cm soil depth between severely burned and unburned sites was 5 °C, which, according to Harden et al. [11], would imply a loss of 10 cm in the organic moss layer thickness in severely burned (CBI > 2) forest areas.

In severely burned forest sites, the complete consumption of the living moss organic layer was strongly associated with warming at the soil surface layer. Measurements showed that soil temperature to 30 cm depth was higher by 8–10 °C compared to unburned forest sites. Below 15 cm soil depth, the temperature of unburned sites dropped gradually to sub-zero (°C) levels by 30 cm depth, while soil temperatures at burned sites remained above 5 °C to 30 cm depth. Our results were similar to those reported by Nossov et al. [24] for fire impacts on forested areas of Yukon Flats and the Yukon-Tanana Uplands—these burns caused a fivefold decrease in surface organic layer thickness, a doubling of water storage in the soil active layer, a doubling of thaw depth, and an increase in soil temperature at the surface (to + 2.1 °C) and at 1 m depth (to + 0.4 °C).

Nearly all of the HBS sites measured during our 2017 field surveys of the Tanana Area Fires had no live surface organic layers remaining. Intense fires during summer of 2015 consumed between 5 and 10 cm of the former live surface organic layer and left behind only a residual dead, charred moss and lichen cover about 3–5 cm deep that had little capacity to insulate the soil layers beneath. We observed that the blackened surface organic layer showed a tendency to be 2–4 °C warmer than the live moss layer under unburned spruce forest strata. These results are consistent with those of Jiang et al. [13] and Brown et al. [7], who reported that post-fire thickness of the soil organic layer and its impact on soil thermal conductivity was the most important factor determining post-fire soil temperatures and thaw depth.

Our total estimate of more than 160 Tg C emitted or lost since 2015 from wildfires in the Yukon-Koyukuk region of Alaska, which included the combined losses from aboveground biomass, surface organic layers, and mineral soil carbon pools, was higher than any previously published fire emission estimate for the forested regions of the state, as listed in Table 1. These previous carbon emission projections for Alaska have included measurements of aboveground (tree) biomass and changes in surface organic layer carbon pools, but have not included potential changes in mineral topsoil carbon pools in severely burned forests. Based on our mineral sampling data from forest soils near Tanana since the 2015 wildfires, which closely match potential carbon loss rates from other forest fire studies in Alaska (Table 5), the contribution of mineral soils to total ecosystem carbon emissions is the highest of the forest strata that are routinely measured.

**Table 5:** Previous estimates of carbon emissions from forest fires in Alaska

Forest stratum	Kg C m^−2^	Fraction consumed			References
		HBS	MBS	LBS	
Aboveground biomass	2.23	0.42	0.34	0.13	Tan et al. [26]
	2.00				Mack et al. [22]
	3.50				Kane and Vogel [16]
Surface organic layer	5.85	0.59	0.39	0.23	Tan et al. [26]
	6.15				Turetsky et al. [28]
	7.70				French et al. [10]
	4.28				Troth et al. [27]
	6.30				Kane and Vogel [16]
Mineral topsoil	10.23				Kane and Vogel [16]^a^

^a^ Estimated based on normalization to soil carbon stocks from 100 years since last disturbance of the sites studied

Additional post-fire losses of between 10 and 15 kg C m^−2^ estimated in our study from thawed mineral soil pools appear to be roughly equivalent to the combined carbon emissions from burned aboveground biomass, live ground cover, and surface organic layers. This potential “mass wasting” and decomposition of the mineral layer (between 10 and 30 cm depth) soil carbon in severely burned areas of the Alaska interior could have occurred at any time between the end of the 2015 fires and the sampling period for this study of July 2017. The soil carbon losses measured in this study were not necessarily emitted during the short 2015 burn period, but instead were likely a consequence of the severe burn conditions that affected these soils following the direct fire emissions of carbon from the nearly complete combustion of aboveground (tree) biomass and in surface organic layers.

## Conclusions

When wildfire areas have an overall percentage of MBS plus HBS areas higher than 60%, as in 2015 for Interior Alaska, vast tracts of forest will be burned deeply into the surface organic layer. This sudden thinning or removal of the moss and soil organic layer will raise post-fire soil temperatures and increase thaw depths, leading to large losses of carbon and nitrogen from mineral soils layers that are much wetter and warmer than the unburned forests nearby. Our results from remote sensing and field measurements in unburned and nearby burned forest sites around Tanana were higher by an average of 8–10 °C compared to unburned forest sites. Combined with nearly unprecedented forest areas severely burned in the Yukon–Koyukuk region of Alaska in 2015, updated total ecosystem fire-related losses of carbon to the atmosphere exceeded most previous estimates for the state by a factor of two, due mainly to the inclusion of potential “mass wasting or decomposition” of mineral soil carbon in the 2 years following these forest fires.
